# Development and validation of the digital well-being scale using university students

**DOI:** 10.3389/fpsyt.2026.1853998

**Published:** 2026-07-17

**Authors:** Joseph Appianing, Frank Quansah, Roger Amoako

**Affiliations:** Department of Educational Foundations, University of Education, Winneba, Ghana

**Keywords:** digital mental health, digital well-being, psychometric validation, scale development, self-determination theory, university students

## Abstract

**Introduction:**

Digital technologies are central to university students' academic, social, and personal lives, offering opportunities for learning and connection while also introducing challenges such as distraction, emotional strain, and difficulties with self-regulation. Despite increasing interest in digital well-being, there is a lack of comprehensive, psychometrically sound instruments specifically designed to assess digital well-being among university students. This study developed and validated the Digital Well-Being Scale (DWS) for use in higher education.

**Methods:**

A cross-sectional survey was conducted among 1,533 undergraduate and postgraduate students from a public university in Ghana. Scale development involved literature review, expert evaluation, pilot testing, and psychometric validation. Exploratory factor analysis (EFA) was used to identify the underlying factor structure, followed by confirmatory factor analysis (CFA) to evaluate construct validity. Reliability, convergent validity, discriminant validity, and measurement invariance across gender were also assessed.

**Results:**

EFA supported a six-factor solution comprising Task Interference, Digital Safety and Responsible Use, Perceived Control and Satisfaction, Digital Life Balance, Emotional Regulation, and Digital Dependence and Frustration. Following CFA-based refinement, a 41-item scale was retained. The six-factor first-order model demonstrated excellent fit to the data and outperformed a higher-order model. The DWS showed satisfactory internal consistency, convergent validity, and discriminant validity across all dimensions. Measurement invariance analyses supported configural and metric invariance across gender, although scalar invariance was only partially supported.

**Discussion:**

The DWS provides a theoretically grounded and psychometrically robust multidimensional measure of digital well-being among university students. The findings suggest that digital well-being encompasses behavioural, emotional, cognitive, self-regulatory, and responsible dimensions of digital engagement rather than representing a single, unidimensional construct. The scale offers a valuable tool for research, screening, and intervention aimed at understanding and promoting digital well-being and digital mental health in higher education settings.

## 1 Introduction

Digital technologies have become a routine part of university life. Students use smartphones, laptops, learning management systems, and social media to help them learn, communicate, and manage their daily lives (1, 2). These tools have made it easier to access information, work with peers, and stay connected to academic activities. At the same time, their constant presence has created a different set of concerns. For many students, being online is now a constant part of their lives. It is continuous, expected, and often difficult to step away from (3, 4).

This matters because digital engagement does not affect students in one simple way. In some cases, it supports learning, autonomy, and connection with others. In other cases, it brings distraction, pressure, and emotional strain (1–3). Students are often managing several streams of digital information at once while also trying to meet academic demands. Notifications, multitasking, and the pressure to stay constantly available can make it harder to concentrate and may gradually increase cognitive overload (5–7). Experimental evidence has shown, for example, that even the mere presence of smartphone notifications can interrupt attention and reduce task performance, including when people try to ignore them (6). Similar concerns have also appeared in studies of digital learning environments, where repeated interruptions and frequent alerts can interfere with concentration and learning (7). These concerns have supported the evolution of the digital well-being into greater focus (4, 8).

Digital well-being is an emerging concept that has been described in different ways across the literature. Existing perspectives generally associate the concept with healthy and balanced technology use, psychological functioning in digital environments, and individuals’ sense of control over their digital experiences (2, 4, 8). However, there is still limited conceptual agreement regarding the precise dimensions that constitute digital well-being, particularly within higher education contexts. Some studies have emphasised digital balance and healthy use, whereas others have focused more narrowly on technostress, problematic technology use, digital competence, or emotional experiences associated with digital engagement (2, 3, 8). This conceptual variation suggests that digital well-being may be broader and multidimensional than is often reflected in existing measures and theoretical discussions. Based on the literature reviewed, digital well-being may collectively be conceptualised as individuals’ capacity to regulate, balance, and adapt to digital engagement in ways that support healthy psychological, emotional, behavioural, social, and daily functioning (2–4, 8).

A number of related concepts have already been studied in this area. These include digital life balance (8, 9), technostress (2, 10), digital competence (11), and problematic smartphone use (3). This body of work has been useful, but much of it has examined only one aspect of students’ digital experience at a time. Studies of technostress have shown how digital demands can become psychologically draining. Research on digital competence has drawn attention to the capacities students need to function effectively in digital environments. Other studies have linked media multitasking with weaker attention regulation and poorer academic outcomes (7, 12). Even so, the broader picture remains somewhat fragmented, as digital well-being is still often studied through these separate dimensions rather than as a broader, multidimensional construct. In many cases, the literature leans heavily either toward negative outcomes such as stress, distraction or dependency or toward more functional concerns such as skill and competence (2, 3, 11). What is often missing is an instrument that captures these different aspects as part of one broader picture of digital well-being.

Some attempts have been made to measure digital well-being more directly as in the case of Arslankara et al. (13) who developed and validated a digital well-being scale for assessing individuals’ interactions with technology. While this represents an important early attempt, some conceptual and contextual limitations exist. First, the scale was developed primarily within a hedonic and eudaimonic conceptual framework that emphasised digital satisfaction, positive emotional experiences, and safe and responsible behaviour in digital environments. Its three dimensions (i.e., Digital Satisfaction, Safe and Responsible Behaviour, and Digital Wellness) largely focused on individuals’ enjoyment of digital technologies, perceived harmony with digital environments, and responsible online conduct. For example, several items assessed positive engagement with technology, including enjoyment of spending time with digital technologies, concern for digital reputation, and happiness associated with social media interactions. While these dimensions are important, they do not adequately capture several increasingly important aspects of digital well-being among university students, including task interference during academic activities, emotional dysregulation, compulsive digital engagement, digital frustration, perceived loss of control, and difficulties maintaining balance between online and offline life.

Second, the scale was developed using heterogeneous sample of general digital technology users aged between 14 and 62 years rather than a specific group such as university students. Consequently, the scale may not sufficiently reflect the distinctive academic, emotional, and self-regulatory challenges associated with intensive digital engagement in higher education settings. In addition, the original study primarily conceptualised digital well-being in relation to digital competence, satisfaction, and wellness, whereas contemporary literature increasingly highlights the multidimensional nature of digital well-being, including attentional functioning, emotional regulation, digital dependence, self-regulation, and balance across digital and non-digital life domains. This issue becomes even more important in African higher education. Patterns of access, digital literacy, and technology use may differ in important ways from those reflected in much of the literature produced in Western settings. Emerging work in Ghana has also pointed to context-specific dimensions of adolescents’ digital experiences, including digital health literacy and related psychosocial outcomes (14).

Another issue worth considering is whether digital well-being is experienced in the same way across different groups of students. Gender, in particular, has received attention in earlier research on digital engagement. Some studies suggest that male students tend to spend more time on entertainment-oriented digital platforms, whereas female students may be more likely to experience emotional pressure linked to social media use (15, 16). Within the Ghanaian context, previous studies have also pointed to gender differences in aspects of students’ digital and psychosocial experiences, including digital health literacy and related coping patterns and well-being outcomes (14, 17). These differences make it important to examine whether digital well-being is understood and measured in a comparable way across gender groups. For this reason, the present study also tested the measurement invariance of the DWS across gender to determine whether the scale functions similarly for male and female students.

It is against this background that the present study developed and validated the Digital Well-Being Scale (DWS) among university students in Ghana. The purpose was to develop a multidimensional instrument for examining how students experience digital engagement in ways that relate to attention, self-regulation, emotional functioning, and day-to-day balance. Thus, the present study contributes to research on digital well-being and related areas of digital mental health by providing a theoretically grounded and empirically tested measure for understanding how digital technologies may shape students’ psychological functioning in everyday life.

## 2 Theoretical framework: self-determination theory

This study is informed by Self-Determination Theory (SDT), which posits that psychological well-being and optimal functioning are supported when three basic psychological needs are met: autonomy, competence, and relatedness (18). Within digital learning environments, these needs may shape how students experience and respond to technology-enhanced academic activities. In this context, autonomy relates to students’ perceived control over their digital engagement, including their ability to regulate technology use and manage digital demands in ways that align with their academic and personal goals. Competence reflects students’ capacity to use digital technologies effectively and confidently in support of learning activities, whereas relatedness concerns students’ sense of social connection and meaningful interaction within digitally mediated environments.

SDT provides a useful framework for understanding digital well-being because digital technologies may either support or frustrate these psychological needs. Digital environments that facilitate self-regulation, effective engagement, and meaningful interaction may contribute positively to students’ well-being, whereas environments characterised by constant interruptions, emotional strain, excessive demands, or diminished control may undermine psychological functioning. From this perspective, digital well-being may be understood as reflecting how students experience, regulate, and adapt to digital engagement within technology-enhanced learning environments. This position helps explain the choice of SDT as the theoretical basis for the DWS because it offers a useful lens for understanding the factors that shape students’ well-being in technology-enhanced learning environments. Relatedness may also be reflected in students’ emotional experiences within digitally mediated environments. Feelings of loneliness, emotional strain, compensatory online engagement, and dependence on digital interactions may partly reflect how students experience social connection, belonging, and interpersonal support in technology-rich contexts. From this perspective, some of the emotional dimensions captured by the DWS may provide insight into how digitally mediated interactions shape students’ broader sense of connectedness and psychological adjustment.

## 3 Materials and methods

### 3.1 Participants

Participants were 1,533 undergraduate and postgraduate students recruited from a large public university in Ghana. The sample comprised 54.8% males and 45.2% females, with ages ranging from 18 years to over 45 years. Students represented a wide range of academic programmes, including education, business, humanities, and the sciences. All participants were enrolled students and regular users of digital technologies for academic and personal purposes. This broad sampling approach was intended to capture variation in digital experiences across academic levels and disciplines, thereby supporting the applicability of the scale within higher education contexts.

### 3.2 Procedure

The study received ethical approval from the University of Education, Winneba Institutional Research Ethics Committee prior to data collection. Participants were informed about the purpose of the study and their right to participate voluntarily, and informed consent was obtained before participation. Data were collected during the 2024/2025 academic year using a structured self-report questionnaire administered through Google Forms. With support from programme administrators and course representatives, the survey link was shared via institutional email lists and academic group communication platforms.

### 3.3 Instrument development and expert reviews

The development of the DWS followed widely recommended procedures for psychological scale development, including item generation, expert review, pilot evaluation, and empirical validation (19). The process began with a review of previous research relating to technostress, digital balance, emotional responses to technology use, and broader discussions of digital well-being. Particular attention was given to studies that described how individuals, and students in particular, experience digital environments in both positive and negative ways.

The initial item pool was informed by research by Elhai et al. (3), Kushlev and Leitao (4), and Wang et al. (2), together with concepts from SDT (18). These sources provided valuable insights into the behavioural and psychological challenges of using technology, including distraction, emotional reactions to digital interactions, and the management of digital engagement. The literature review also guided the identification of key behavioural, emotional, cognitive, and self-regulatory experiences that appeared consistently across discussions of digital well-being and related constructs.

Drawing on these insights, several broad thematic areas were identified that appeared to capture the major dimensions of students’ digital experiences. These themes served as guiding categories during the item-generation process rather than predetermined subscales. Examples of these thematic areas included attentional interference during academic activities, emotional responses to digital engagement, regulation of screen use, perceptions of control over digital behaviour, responsible online practices, and balance between online and offline activities. Based on these themes, an initial pool of 48 items was generated. The items were written to reflect common situations encountered by university students when using digital technologies for both academic and personal purposes. Care was taken to ensure that the items were concise, contextually relevant to students’ experiences, and written language that could be easily understood by university students from different academic backgrounds.

Following the generation of the initial items, three subject matter experts, each possessing pertinent expertise in psychometrics, measurement, and educational psychology, conducted a review. The experts independently evaluated the items for clarity, conceptual relevance, representativeness of the intended thematic areas, appropriateness for the target population, and potential overlap or redundancy across items. Particular attention was given to whether the items adequately reflected the broader construct of digital well-being as conceptualised in the study. These experts were asked to indicate whether each item should be retained, revised, or removed. Items were generally retained when there was agreement among the reviewers that they were conceptually relevant, clearly worded, and appropriately aligned with the intended thematic area. Items identified as unclear, repetitive, overly broad, or insufficiently representative of the construct were revised based on the experts’ recommendations. In cases where experts provided differing opinions regarding item wording or relevance, the research team reviewed the feedback collectively and resolved disagreements through discussion, with decisions guided by conceptual alignment with the construct and the overall clarity of the item.

The feedback provided by these experts facilitated the refinement of several item wordings. For instance, one item originally phrased as “I can’t control my phone use” was revised to “It is difficult for me to stop using my phone, even when I have important things to do, “ which provided a clearer description of the behaviour being measured. No items were discarded at this stage because the reviewers agreed that the initial pool represented the intended conceptual coverage of the construct, although several items required refinement to improve clarity and contextual relevance. The expert review process served as an important step in establishing preliminary evidence of content validity by helping ensure that retained items were relevant, representative, and comprehensible prior to empirical testing.

After the expert-based revisions, we pretested the survey items with a small group of university students prior to the main online survey to assess clarity and relatability. The pilot focused on whether students clearly understood the wording of the items, considered them relevant to their digital experiences, and encountered any difficulties interpreting the response options. Their feedback informed additional refinements. For example, the item “I ensure my passwords are regularly updated and complex” was revised to “I make sure my passwords are strong and update them often, “ as students indicated that the original wording sounded overly formal. Minor wording revisions were also made to improve readability, reduce ambiguity, and enhance the natural flow of the statements.

Students rated each item on a four-point Likert scale ranging from 1 (strongly disagree) to 4 (strongly agree), indicating how well each statement reflected their own experiences. A four-point response format was intentionally adopted to reduce the tendency for respondents to select neutral responses and to encourage clearer expression of agreement or disagreement. Negatively worded items were reverse-scored so that higher scores consistently reflected more favourable digital well-being across the scale.

### 3.4 Statistical analyses

All statistical analyses in the present study were conducted using JASP, following distinct but interrelated stages of data analysis. The first stage involved preliminary data screening for missing values, outliers, and normality. Basic descriptive statistics (frequencies, percentages, means, standard deviations, and minimum-maximum values) were then used to screen and clean the dataset.

We first conducted EFA (minimum residual extraction with oblimin rotation) after confirming sampling adequacy (KMO) and sphericity (Bartlett’s test). Factor retention was informed by parallel analysis, the scree plot, and conceptual interpretability; items with weak or problematic cross-loadings were reviewed. We then used CFA to test the EFA-derived structure and evaluated model fit using standard indices (Comparative Fit Indices, CFI; Tucker-Lewis Index, TLI; Root Mean Square Error of Approximation, RMSEA; Standardised Root Mean Residual, SRMR). Reliability (α, ω, CR), convergent validity Average Variance Extracted (AVE), and discriminant validity (Fornell–Larcker and Heterotait-Monotrait Ratio (HTMT) were examined, followed by multigroup CFA to test gender invariance (configural, metric, scalar) using changes in CFI and RMSEA.

## 4 Results

### 4.1 Demographic characteristics of the students

As shown in **Table 1**, the sample comprised 1, 533 students, with male students outnumbering female students (54.8% vs. 45.2%). The largest proportion of students fell within the age range of 21 to 25 years (30.1%), followed by the 31- to 35-year-old category (26.5%). The sample was dominated by undergraduates (84.6%), with about 38.9% constituting first-year students. With regard to digital behaviours, an overwhelming majority of students reported smartphones (90.4%) as their main device. About 90% indicated that they accessed the internet via personal mobile data. Daily screen use was also high, with 58.6% reporting more than 3 hours of screen time per day. Only a small proportion reported having formal digital literacy training (13%).

**Table 1:** Demographic characteristics of the students.

Variable	Levels	Frequency (n)	Percentage (%)
Sex	Male	840	54.8
	Female	693	45.2
Age	15–20 years	241	15.7
	21–25 years	462	30.1
	26–30 years	243	15.9
	31–35 years	407	26.5
	36 years and above	180	11.7
Academic Level	Level 100	597	38.9
	Level 200	273	17.8
	Level 300	220	14.4
	Level 400	207	13.5
	Postgraduate	236	15.4
Average Daily Screen Time	Less than 1 hour	223	14.5
	1–2 hours	521	34.0
	3–4 hours	377	24.6
	5–6 hours	276	18.0
	More than 6 hours	136	8.9
Primary Device Used	Smartphone	1, 386	90.4
	Laptop/Desktop	123	8.0
	Tablet/iPad	24	1.6
Primary Internet Access Means	Personal mobile data	1, 380	90.0
	Wi-Fi (home or hostel)	100	6.5
	Campus internet	50	3.3
	Cybercafé/public access	3	0.2
Digital Literacy Training	Yes	199	13.0
	No	1, 334	87.0

### 4.2 Factor structure of the digital well-being scale: exploratory factor analysis

Sample adequacy: Prior to performing the EFA, preliminary analyses were conducted to examine whether the obtained data were appropriate for the analysis. The KMO measure of sampling adequacy yielded an overall value of 0.878, indicating that the data were suitable for factor analysis (20). Examination of the item-level KMO values further revealed values ranging from 0.686 to 0.931, suggesting that the inter-item correlations were adequate. Bartlett’s test of sphericity was also significant, χ^2^ (1128) = 9583, p < 0.001, confirming that the relationships among the indicators were sufficiently strong for EFA (21).

Model fit: To determine the adequacy of the factor solution produced by the analysis, model-fit indicators were examined. Overall, the fit indices were acceptable, with RMSEA = 0.044 (CI [0.041, 0.048]), χ^2^ (813) = 1632, p < 0.001, BIC = -3436, TLI = 0.864, and cumulative variance explained of 42.2%, which is acceptable for a multifactor scale (22). Although the chi-square test was significant, this is unsurprising given the large sample size. The slightly lower TLI was interpreted with caution at this exploratory stage.

**Figure 1:**
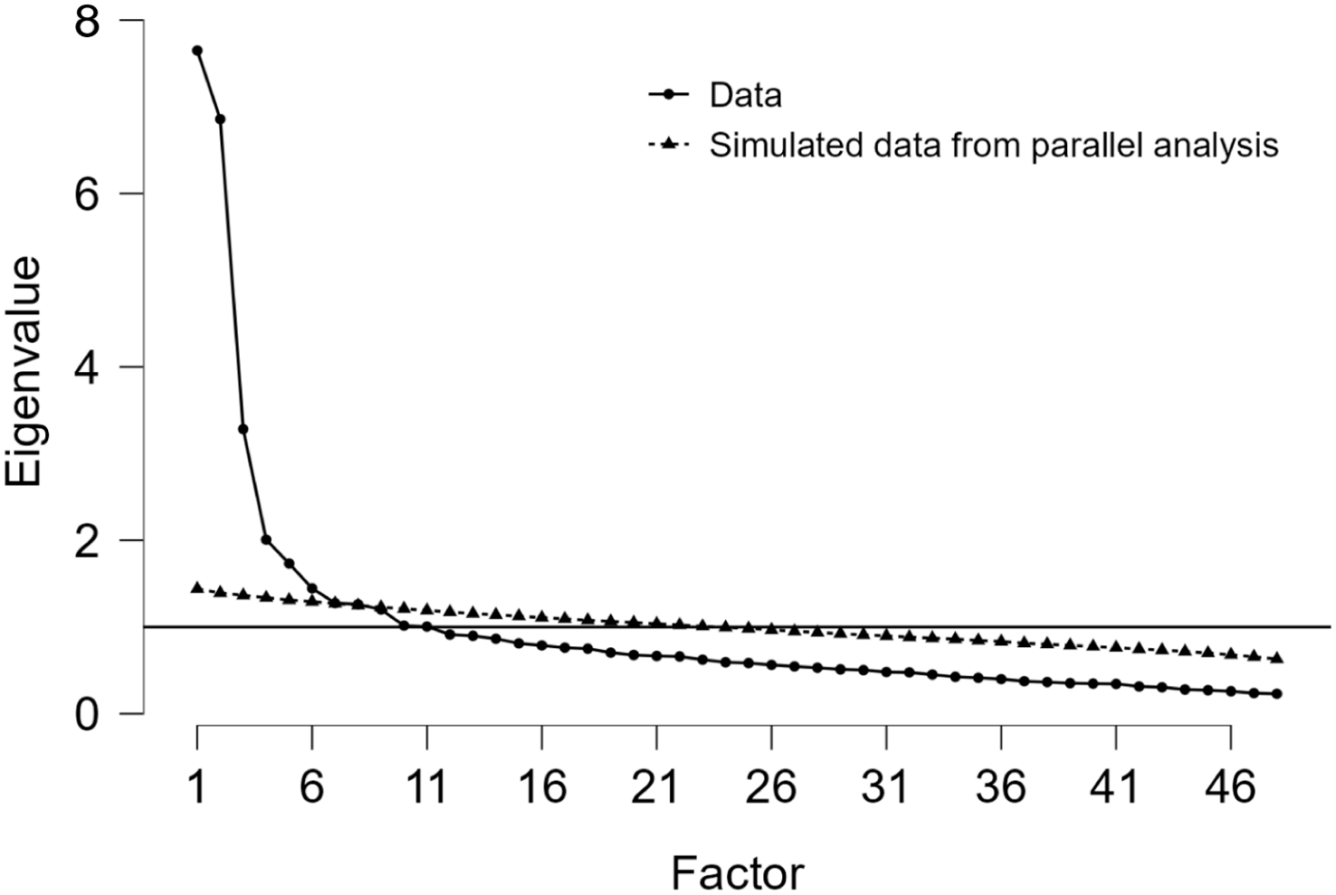
Scree plot with parallel analysis.

Structure: Following confirmation of sampling adequacy and acceptable overall fit, the factor structure of the digital well-being scale was examined using the scree plot, parallel analysis, and eigenvalue criteria. Theoretical interpretability of the extracted factors was evaluated with reference to the factor-loading matrix. Inspection of the scree plot and the results of the parallel analysis (see **Figure 1**) showed that the eigenvalues derived from the observed data exceeded those from the random simulations up to the sixth factor, supporting a six-factor structure.

The six factors together explained 42.2% of the total variance, with eigenvalues of 6.93, 5.91, 2.44, 1.22, 0.85, and 0.58. Although the initial output suggested a possible seventh factor, it did not hold up on closer inspection because its eigenvalue was minimal and it was defined by only two overlapping items. The six-factor model was therefore retained because it offered a clearer and more interpretable structure. The 48 items loaded on the six factors at values above 0.30 (23): Factor 1 (13 items), Factor 2 (8 items), Factor 3 (7 items), Factor 4 (9 items), Factor 5 (7 items), and Factor 6 (4 items) (see **Table 2**).

**Table 2:** EFA loadings, CFA standardized loadings, standard error and p-values of indicators.

No.	Factor/Item	EFA Loading	CFA Std. Estimate (β)	SE	p
Factor 1: Task interference					
1.	I often spend more time online than I meant to.	.424	.521	.038	<.001
2.	It is difficult for me to stop using my phone, even when I have important things to do.	.468	.628	.042	<.001
**3.**	**I think I get too many texts, notifications, and SMS.**	**.326**	**.455**	**.026**	**<.001**
**4.**	**How well I can sleep or rest depends on how I utilise digital devices.**	.387	.434	.042	<.001
5.	I miss out on real life things because I spend too much time online.	.521	.611	.039	<.001
6.	I don’t like how much time I spend online.	.431	.458	.037	<.001
7.	My phone or social media gets in the way when I am studying.	.525	.632	.035	<.001
8.	I go on social media or text while I’m in class or having tutorials.	.586	.594	.038	<.001
9.	Spending too much time on social media affects my academic work.	.602	.621	.039	<.001
10.	I postpone my homework to watch movies or browse online.	.709	.724	.036	<.001
11.	When I’m online, I often forget about my studies because I lose sight of time.	.806	.729	.035	<.001
12.	I often find myself switching between studying and checking my phone or browsing online.	.682	.711	.037	<.001
13.	I cannot concentrate on my studies when my phone is nearby.	.714	.747	.037	<.001
Factor 2 – Digital safety and responsible use					
14.	I am careful about the private information I provide online.	.766	.792	.031	<.001
15.	I often look at the privacy settings on my social media accounts.	.629	.645	.033	<.001
16.	I do not worry about communicating to individuals online.	.424	.501	.037	<.001
17.	I have seen things online that are harmful or rude.	.682	.646	.036	<.001
18.	I know how to report or block threats and harassment that happen online.	.612	.603	.037	<.001
19.	I do not click on links or communications that look suspicious.	.759	.761	.032	<.001
20.	I make sure my passwords are strong and update them often.	.711	.753	.032	<.001
21	I think carefully before accepting messages or friend requests from people I do not know.	.802	.814	.029	<.001
Factor 3 – Perceived control and satisfaction					
22.	I like how I use digital tools every day.	.743	.723	.031	<.001
23.	I believe I can choose how to use digital tools.	.516	.701	.030	<.001
24.	What I do on the internet helps me reach my goals and become a better person.	.675	.644	.032	<.001
25.	I believe that the amount of time I spend online right now is excellent for my health.	.585	.538	.039	<.001
26.	I choose digital tools on purpose that make my life better.	.735	.705	.031	<.001
27.	I think about how digital devices influence my behaviours and prioritise accordingly.	.459	.569	.033	<.001
28.	I feel like I can adjust my digital practices when necessary.	.485	.604	.031	<.001
Factor 4 – Digital life balance					
29.	I reduce my screen time on purpose so I can do other things.	.395	.385	.037	<.001
30.	I decide when and how I use my digital devices without any worry.	.453	.509	.036	<.001
**31.**	**I use tools or apps to help me keep track of how much time I spend online.**	.406	.323	.043	<.001
**32.**	**I think about how much time I spend on screens every day to help me stay focused and get more done.**	.373	.353	.038	<.001
33.	I use digital devices when I am eating or talking to others.	.436	.476	.043	<.001
34.	I always set aside time to turn off all my digital devices.	.436	.413	.041	<.001
35.	I often spend time away from the internet to attend to important activities.	.442	.619	.035	<.001
36.	I have a decent control between my online and offline activities.	.453	.705	.033	<.001
37.	I can quickly turn off my devices when I need to.	.405	.553	.039	<.001
Factor 5 – Emotional regulation					
38.	When I spend too much time online, I feel lonely.	.570	.538	.040	<.001
39.	I feel bad mentally when I do not use my phone or computer for a long.	.535	.555	.044	<.001
40.	I do not like how social media makes me feel.	.592	.577	.044	<.001
**41.**	**I feel emotionally drained after spending a lot of time in front of a screen.**	**.419**	**−.053**	**.060**	**.441**
42.	I do things online to keep myself from being bored or feeling awful.	.434	.580	.046	<.001
43.	There are certain times of the day when I do not use my digital device at all.	.419	.467	.051	<.001
44.	When I reduce my screen time, I feel more present and mindful.	.323	.431	.044	<.001
Factor 6 – Digital Dependence and Frustration					
45.	I get anxious when I cannot get on the internet or use my phone.	.357	.471	.045	<.001
46.	When my internet is slow or does not operate, I become irritated quickly.	.572	.686	.047	<.001
47.	I use the internet to cope with boredom or negative feelings.	.507	.772	.053	<.001
**48.**	**I like talking to people in person more than online.**	**−.322**	**−.179**	**.051**	**.001**

NB: Items in bold were removed.

### 4.3 Construct validity of the DWS: confirmatory factor analysis

We conducted a two-stage confirmatory factor analysis. The first stage examined the contribution of each indicator in light of the EFA results to determine whether any items should be removed. The second stage re-estimated the model with the retained items to evaluate indicator strength and to test whether the scale was better represented as a first-order or higher-order structure.

#### 4.3.1 Phase 1: item evaluation and comparison with EFA output

Overall, the CFA output was broadly consistent with the six-factor model extracted from the EFA. Most items showed strong standardised loadings and significant parameters, although some indicators demonstrated weak or unstable loadings and were therefore reviewed during model refinement. Within Task Interference, three items (“I think I get too many texts, notifications, and SMS.”, “How well I can sleep, or rest depends on how I utilise digital devices.”, “I don’t like how much time I spend online.”) showed persistently weaker loadings across the EFA and CFA and were excluded from the final retained set. All 8 items in Digital Safety and Responsible Use were retained because they showed good convergence across analyses (EFA = 0.424 - 0.802; CFA = 0.501 - 0.814). Likewise, all 7 items in Perceived Control and Satisfaction were retained because of their consistently strong performance (EFA = 0.459 - 0.743; CFA = 0.538 - 0.723).

After careful consideration of the items under Digital Life Balance, two items were removed because of persistently low factor loadings across both the EFA (0.404 and 0.373, respectively) and the CFA (0.323 - 0.353, respectively): “I use tools or apps to help me keep track of how much time I spend online” and “I think about how much time I spend on screens every day to help me stay focused and get more done.” Although three other items in this factor showed comparatively lower loadings, they were retained to preserve conceptual coverage, as recommended by the expert reviewers.

Within Emotional Regulation, one item (“I feel emotionally drained after spending a lot of time in front of a screen”) showed a non-significant loading (EFA = 0.419, CFA = -0.53, p = 0.441) and was removed. Two other items showed weak to moderate loadings (“There are certain times of the day when I do not use my digital device at all” and “When I limit my screen time, I feel more present and mindful”), but these were retained on theoretical and conceptual grounds. In Digital Dependence and Frustration, one of the four initial items (“I like talking to people in person more than online”) showed an inconsistent loading pattern and was therefore removed. Thus, across the refinement process, six items were excluded overall, yielding the final 41-item solution used in Phase 2.

Following a thorough review of the selected items and expert panel feedback, the subscales were defined and described as follows:

Factor 1: Task Interference – This factor reflects the extent to which digital technologies disrupt students’ academic tasks and goal-directed activities. It encompasses attentional distraction, difficulties disengaging from digital content, and the tendency to postpone important responsibilities because of digital engagement.
Factor 2: Digital Safety and Responsible Use – This factor reflects students’ awareness of digital risks and their engagement in safe, ethical, and responsible digital practices. It includes behaviours related to privacy protection, critical evaluation of online information, and responsible participation in digital environments.
Factor 3: Perceived Control and Satisfaction – This dimension reflects students’ perceived autonomy, intentionality, and satisfaction in their use of digital technologies. It captures the extent to which students feel able to regulate their digital engagement in ways that align with their academic and personal goals.
Factor 4: Digital Life Balance – This factor reflects students’ ability to maintain a healthy balance between digital engagement and offline activities. It captures effective management of competing academic, social, and personal demands across digital and non-digital contexts.
Factor 5: Emotional Regulation – This factor reflects emotional experiences and coping responses associated with digital engagement. It includes feelings such as loneliness, emotional discomfort linked to digital use, reliance on online activities to cope with boredom, or negative emotions, and the ability to regulate their emotional experiences through healthier digital habits.
Factor 6: Digital Dependence and Frustration – This factor reflects the emotional strain and perceived reliance associated with digital technology use. It encompasses anxiety or irritation when disconnected, frustration arising from digital disruptions, and a tendency to rely on digital technologies to manage boredom or discomfort.

#### 4.3.2 Phase 2: model re-estimation with retained items

We re-estimated the CFA using the retained 41 items and compared two alternatives: a six-factor first-order model and a six-factor second-order model. The first-order model showed substantially better fit to the data (χ^2^(804) = 6749.86, p < 0.001; CFI = 0.977; TLI = 0.961; RMSEA = 0.035; SRMR = 0.017) than the second-order model (CFI = 0.871; TLI = 0.876; RMSEA = 0.085; SRMR = 0.107). These results suggest that the six dimensions of digital well-being are better understood as related but distinct domains rather than as indicators of a single higher-order factor. Consequently, subsequent analyses focused on the first-order six-factor model.

As shown in **Table 3** and **Supplementary Figure 1** (see **supplementary information**), all retained indicators loaded sufficiently and significantly on their respective dimensions, with standardised loadings ranging from 0.494 to 0.824 (24). The Task Interference subscale, for example, demonstrated strong loadings (λ = 0.519 - 0.744). The other domains showed a similar pattern (Digital Safety and Responsible Use, λ = 0.494 - 0.814; Perceived Control and Satisfaction, λ = 0.537 - 0.723; Digital Life Balance, λ = 0.562 - 0.824; Emotional Regulation, λ = 0.503 - 0.773; Digital Dependence and Frustration, λ = 0.581 - 0.746). Internal consistency across the factors was adequate, with Task Interference showing the highest reliability coefficients (α = 0.880, ω = 0.881, CR = 0.880). Although a few retained indicators demonstrated comparatively modest factor loadings, they were preserved because they contributed important conceptual breadth to the multidimensional representation of digital well-being. Retaining these indicators helped ensure that the scale captured the broader behavioural, emotional, cognitive, and self-regulatory experiences associated with students’ digital engagement rather than narrowing the construct to only highly homogeneous experiences.

**Table 3:** Standardised factor loadings, variances, and reliability estimates for the six-factor digital well-being model.

No.	Factor/Item	Factor Loading (λ)	p	Variance (λ^2^)
Factor 1 – Task interference (α = 0.880, ω = 0.881, CR = 0.880, AVE = 0.43)				
TIF1	I often spend more time online than I meant to.	0.519	<.001	0.269
TIF2	It is difficult for me to stop using my phone, even when I have important things to do.	0.626	<.001	0.392
TIF3	I miss out on real life things because I spend too much time online.	0.595	<.001	0.354
TIF4	My phone or social media gets in the way when I am studying.	0.628	<.001	0.394
TIF5	I go on social media or text while I’m in class or having tutorials.	0.603	<.001	0.364
TIF6	Spending too much time on social media affects my academic work.	0.614	<.001	0.377
TIF7	I postpone my homework to watch movies or browse online.	0.731	<.001	0.535
TIF8	When I am online, I often forget about my studies because I lose sight of time.	0.733	<.001	0.537
TIF9	I often find myself switching between studying and checking my phone or browsing online.	0.719	<.001	0.517
TIF10	I cannot concentrate on my studies when my phone is nearby.	0.744	<.001	0.554
Factor 2 – Digital safety and responsible use (α = 0.876, ω = 0.881, CR = 0.880, AVE = 0.49)				
DSR1	I am careful about the private information I provide online.	0.792	<.001	0.627
DSR2	I often look at the privacy settings on my social media accounts.	0.645	<.001	0.416
DSR3	I do not worry about communicating to individuals online.	0.494	<.001	0.244
DSR4	I have seen things online that are harmful or rude.	0.646	<.001	0.418
DSR5	I know how to report or block threats and harassment that happen online.	0.604	<.001	0.365
DSR6	I do not click on links or communications that look suspicious.	0.762	<.001	0.581
DSR7	I make sure my passwords are strong and update them often.	0.753	<.001	0.567
DSR8	I think carefully before accepting messages or friend requests from people I do not know.	0.814	<.001	0.663
Factor 3 – Perceived control and satisfaction (α = 0.826, ω = 0.830, CR = 0.830, AVE = 0.42)				
PCS1	I like how I use digital tools every day.	0.723	<.001	0.523
PCS2	I believe I can choose how to use digital tools.	0.702	<.001	0.493
PCS3	What I do on the internet helps me reach my goals and become a better person.	0.645	<.001	0416
PCS4	I believe that the amount of time I spend online right now is excellent for my health.	0.537	<.001	0.288
PCS5	I choose digital tools on purpose that make my life better.	0.705	<.001	0.497
PCS6	I think about how digital devices influence my behaviours and prioritise accordingly.	0.567	<.001	0.322
PCS7	I feel like I can adjust my digital practices when necessary.	0.605	<.001	0.366
Factor 4 – Digital life balance (α = 0.720, ω = 0.727, CR = 0.860, AVE = 0.48)				
DLB1	I reduce my screen time on purpose so I can do other things.	0.562	<.001	0.316
DLB2	I decide when and how I use my digital devices without any worry.	0.689	<.001	0.475
DLB3	I use digital devices when I am eating or talking to others.	0.658	<.001	0.433
DLB4	I always set aside time to turn off all my digital devices.	0.577	<.001	0.333
DLB5	I often spend time away from the internet to attend to important activities.	0.734	<.001	0.539
DLB6	I have a decent control between my online and offline activities.	0.824	<.001	0.679
DLB7	I can quickly turn off my devices when I need to.	0.773	<.001	0.598
Factor 5 – Emotional regulation (α = 0.723, ω = 0.740, CR = 0.883, AVE = 0.45)				
ERG1	When I spend too much time online, I feel lonely.	0.743	<.001	0.552
ERG2	I feel bad mentally when I do not use my phone or computer for a long.	0.761	<.001	0.579
ERG3	I do not like how social media makes me feel.	0.568	<.001	0.323
ERG4	I do things online to keep myself from being bored or feeling awful.	0.773	<.001	0.597
ERG5	There are certain times of the day when I do not use my digital device at all.	0.503	<.001	0.253
ERG6	When I reduce my screen time, I feel more present and mindful.	0.639	<.001	0.408
Factor 6 – Digital dependence and frustration (α = 0.695, ω = 0.704, CR = 0.710, AVE = 0.46)				
DDF1	I get anxious when I cannot get on the internet or use my phone.	0.581	<.001	0.338
DDF2	When my internet is slow or does not operate, I become irritated quickly.	0.691	<.001	0.478
DDF3	I use the internet to cope with boredom or negative feelings.	0.742	<.001	0.551

The AVE values ranged from 0.420 to 0.490 across the six factors. Although these estimates fell marginally below the conventional benchmark of 0.50 suggested by Fornell and Larcker (25), convergent validity was considered acceptable for several reasons. First, all factors demonstrated satisfactory reliability indices and substantial standardised factor loadings, indicating adequate shared variance among the indicators. Fornell and Larcker (25) noted that lower AVE values may not necessarily indicate problematic measurement when composite reliability and factor loadings remain adequate, as such findings may reflect acceptable construct representation rather than excessive measurement error. Second, the DWS was intentionally developed to capture broad and multidimensional aspects of digital well-being, including behavioural, emotional, cognitive, self-regulatory, and responsible dimensions of digital engagement. Because these domains reflect conceptually diverse but related psychological experiences, slightly lower AVE estimates may reasonably occur due to the breadth and complexity of the construct rather than poor indicator quality. Third, the overall CFA results demonstrated strong model fit, further supporting the adequacy of the retained measurement structure despite the marginally lower AVE estimates.

### 4.4 Discriminant validity

The discriminant validity evidence was examined using the inter-factor correlations, the Fornell-Larcker criterion (25), and the Heterotrait-Monotrait (HTMT) ratio of correlations (26). As shown in **Table 4**, the square roots of the AVE values for each construct were greater than the corresponding inter-construct correlations, supporting discriminant validity among the six dimensions. For example, the Task Interference domain correlated strongly with the Digital Dependence and Frustration domain (r = 0.570), yet the square root of its AVE was higher than this correlation (√AVE = 0.660). A similar pattern was observed across the remaining factors. The HTMT values were also below the conservative threshold of 0.85 suggested by Kline (27), ranging from 0.380 to 0.690.

**Table 4:** Discriminant validity BASED on Fornell-Larcker criterion and HTMT ratio of correlation.

ID.	Dimensions	√AVE	HTMT (Max. value)
TIF	Task Interference	0.660	0.570
DSR	Digital Safety and Responsible Use	0.700	0.520
PCS	Perceived Control and Satisfaction	0.650	0.690
DLB	Digital Life Balance	0.690	0.690
ERG	Emotional Regulation	0.670	0.380
DDF	Digital Dependence and Frustration	0.680	0.570

TIF, Task Interference; DSR, Digital Safety and Responsible Use; PCS, Perceived Control and Satisfaction; DLB, Digital Life Balance; ERG, Emotional Regulation; DDF, Digital Dependence and Frustration.

The inter-factor correlations among the six dimensions are presented in **Table 5**. Overall, the correlation coefficients ranged from weak to moderately strong, suggesting that the dimensions were related but empirically distinguishable. The strongest positive association was observed between Perceived Control and Satisfaction, and Digital Life Balance (r= 0.685). Task Interference also showed a relatively strong positive correlation with Digital Dependence and Frustration (r= 0.568). Negative correlations were observed between Task Interference and both Perceived Control and Satisfaction (r= -0.144) and Digital Life Balance (r= -0.189). The pattern of correlation supported the related yet distinct nature of the six dimensions.

**Table 5:** Inter-factor correlation among the six dimensions of DWB.

Construct	TIF	DSR	PCS	DLB	ERG	DDF
TIF	1.00					
DSR	0.03	1.00				
PCS	-0.14	0.52	1.00			
DLB	-0.19	0.47	0.69	1.00		
ERG	0.34	0.04	0.16	0.38	1.00	
DDF	0.57	0.30	0.17	0.13	0.27	1.00

TIF, Task Interference; DSR, Digital Safety and Responsible Use; PCS, Perceived Control and Satisfaction; DLB, Digital Life Balance; ERG, Emotional Regulation; DDF, Digital Dependence and Frustration.

### 4.5 Gender measurement invariance

We examined gender measurement invariance for the six-factor DWS to determine whether the instrument functioned similarly for male and female students. Multi-group CFA was conducted following the sequential procedure proposed by Byrne (28). Three nested models were tested: configural, metric, and scalar invariance.

As shown in **Table 6**, configural invariance was established [χ^2^(1608) = 8,279.879, p < 0.001, CFI = 0.952, TLI = 0.928, RMSEA = 0.009, SRMR = 0.011], indicating that male and female students conceptualised digital well-being in a comparable six-factor structure. Metric invariance was also supported [χ^2^(1644) = 8,448.76, p < 0.001, CFI = 0.945, TLI = 0.929, RMSEA = 0.009, SRMR = 0.015], demonstrating comparable factor loadings across gender (29). The scalar model showed a further decline in fit [χ^2^(1680) = 8,725.085, p < 0.001, CFI = 0.933, TLI = 0.924, RMSEA = 0.009, SRMR = 0.013]. Although the change in RMSEA and SRMR was negligible, the decrease in CFI (ΔCFI = 0.012) slightly exceeded the conventional guideline, so scalar invariance should be interpreted cautiously (30).

**Table 6:** Gender measurement invariance.

Model	CFI	TLI	RMSEA	SRMR	ΔCFI
Configural invariance	0.952	0.928	0.009	0.011	—
Metric invariance	0.945	0.929	0.009	0.015	−0.007
Scalar invariance	0.933	0.924	0.009	0.013	−0.012

## 5 Discussion

This study developed and validated the DWS as a multidimensional measure of university students’ digital experiences. The findings supported a six-factor structure made up of Task Interference, Digital Safety and Responsible Use, Perceived Control and Satisfaction, Digital Life Balance, Emotional Regulation, and Digital Dependence and Frustration. These dimensions suggest that digital well-being is not a single, uniform state but rather, it reflects several connected ways in which students experience, manage, and respond to digital technologies in their academic and everyday lives. This finding is important because digital technologies are now woven into how students learn, communicate, organise their work, and regulate their attention. The DWS therefore provides a way of examining digital engagement not simply as technology use, but as part of students’ broader functioning within learning environments. In this sense, the scale adds to existing work by bringing together dimensions that earlier studies have often examined separately, including distraction, emotional strain, perceived control, balance, and responsible use. The findings also support the view that digital engagement can have both supportive and disruptive implications for students’ well-being, depending on how it is experienced and managed (2, 4, 8, 13).

Relatedly, the six interrelated dimensions identified provide a more focused conceptualisation of digital well-being. As earlier stated, existing literature has commonly described digital well-being in terms of healthy technology use, balance between online and offline life, and the extent to which digital engagement supports psychological well-being and personal functioning (4, 8, 13). While these perspectives have been useful, they have often conceptualised digital well-being primarily as a general state of healthy or balanced technology use. The findings of the present study suggest a broader conceptualisation in which digital well-being reflects individuals’ capacity to function adaptively within digital environments across behavioural, emotional, cognitive, self-regulatory, and ethical domains of experience. From this perspective, digital well-being is not only reflected in balanced or satisfying technology use, but also in individuals’ ability to manage digital interference, maintain emotional regulation, exercise control over digital engagement, participate responsibly in online spaces, sustain healthy integration between online and offline life, and avoid excessive dependence and frustration associated with digital technologies.

Some of the identified dimensions reflect the more difficult side of digital life. Task Interference, Digital Dependence and Frustration, and Emotional Regulation point to the ways digital environments can place pressure on attention, self-regulation, and emotional stability. In technology-rich learning settings, students are often required to move across multiple digital platforms while also dealing with notifications, messages, and online distractions. Under such conditions, it is not surprising that digital engagement may begin to interfere with concentration, heighten frustration, or contribute to emotional strain. The presence of these dimensions in the scale reinforces the idea that digital well-being is closely tied to how students cope with the demands of constant connectivity and digital interruption (2, 3, 6, 7).

In contrast to the dimensions reflecting digital strain and disruption, the emergence of Perceived Control and Satisfaction, Digital Life Balance, and Digital Safety and Responsible Use highlight the adaptive dimensions of digital well-being. These dimensions suggest that digital well-being is not solely concerned with avoiding harm or reducing problematic technology use. Rather, it also involves students’ capacity to regulate their digital engagement, maintain balance between online and offline activities, and participate in digital environments in informed and responsible ways. This interpretation aligns with broader well-being literature which suggests that psychological well-being is supported when individuals are able to maintain balance, exercise self-regulation, and engage meaningfully with their environments (32, 33). Within digital contexts, these capacities may help students experience technology as supportive rather than psychologically burdensome, consistent with emerging views of digital well-being as a dynamic and context-dependent construct (34). This broader perspective recognises that digital well-being encompasses both vulnerabilities and strengths, rather than merely the absence of negative outcomes associated with technology use (8, 9, 11).

The findings of the study can also be interpreted through the lens of SDT, which emphasises autonomy, competence, and relatedness as central psychological needs underlying well-being (18, 31). The dimensions of the DWS may be interpreted as reflecting these psychological needs within digital environments. Perceived Control and Satisfaction, and Digital Life Balance primarily relate to autonomy because they reflect students’ ability to regulate and balance their digital engagement intentionally. Digital Safety and Responsible Use capture competence through students’ confidence and effectiveness in navigating digital environments responsibly. Task Interference, Emotional Regulation, and Digital Dependence and Frustration reflect ways in which digital engagement may undermine self-regulation, attentional functioning, and emotional stability, thereby frustrating autonomy and competence needs. Although relatedness did not emerge as a distinct factor, aspects of emotional strain and constant digital connectedness suggest that students’ social experiences within digital environments may still shape their broader sense of connection and well-being. The stronger fit of the first-order model further supports the view that these dimensions represent related but distinct aspects of psychological functioning in digital contexts rather than manifestations of a single undifferentiated experience.

The inter-factor correlations provide additional evidence regarding the multidimensional nature of digital well-being. Although the six dimensions were empirically distinguishable, several moderate associations were observed, suggesting that they represent interconnected aspects of students’ digital experiences rather than entirely independent constructs. In particular, the strong positive association between Perceived Control and Satisfaction and Digital Life Balance suggests that students who experience greater autonomy and satisfaction in their digital engagement are also more likely to maintain healthier boundaries between digital and non-digital activities. This finding aligns with existing well-being literature, which emphasises self-regulation, balance, and perceived control as important foundations of psychological well-being and healthy functioning (32, 33). The outcome also supports the proposition that digital well-being extends beyond the absence of problematic technology use and includes adaptive capacities that enable individuals to engage with digital technologies in purposeful and balanced ways (34).

The correlation pattern also highlights the close relationship between disruptive and emotionally taxing forms of digital engagement. The relatively strong association between Task Interference and Digital Dependence and Frustration suggests that difficulties managing digital distractions may coexist with feelings of frustration, anxiety when disconnected, and greater reliance on digital technologies to cope with boredom or discomfort. Conversely, the negative associations between Task Interference and both Perceived Control and Satisfaction and Digital Life Balance indicate that disruptive digital engagement may undermine students’ sense of control and their ability to maintain balanced technology use. These observations are consistent with contemporary well-being perspectives, which view well-being as a dynamic process involving the successful management of environmental demands and personal resources rather than merely the absence of distress (32, 35). They further support emerging conceptualisations of digital well-being as reflecting both vulnerabilities and strengths within individuals’ interactions with digital environments (34).

The scale also functioned in a broadly similar way for male and female students at the configural and metric levels, which supports its use for examining the structure of digital well-being and the relationships among the dimensions across gender groups. The establishment of metric invariance further suggests that the factor loadings operated similarly for male and female students, indicating that the dimensions carried comparable psychological meaning across groups. However, support for scalar invariance was weaker, suggesting that some item intercepts may differ between males and females. This implies that observed group differences in scores may not always reflect equivalent differences in the underlying latent constructs. Consequently, comparisons involving latent means or observed scale scores across gender groups should be interpreted with caution (29, 30). Within both Ghanaian and broader international contexts, previous research has shown that male and female students may differ in patterns of digital engagement, emotional experiences associated with technology use, online participation, and coping responses in digital environments (14–17). Such differences may partly influence how certain digital well-being experiences are interpreted or endorsed across gender groups, even when the overall construct structure remains comparable. These findings nevertheless provide encouraging evidence that the DWS captures a broadly comparable multidimensional structure of digital well-being across male and female students, while also indicating the need for further examination of potential item-level differences across groups in future research.

### 5.1 Practical implications

The DWS has practical value for both research and applied work in digital mental health. For researchers, it provides a structured and psychometrically grounded way of examining how different aspects of digital engagement relate to psychological well-being, emotional strain, and self-regulation in university students. Rather than treating digital engagement as a single broad construct, the scale allows researchers to examine how attentional interference, emotional regulation, digital dependence, and perceived control interact in students’ everyday experiences. This multidimensional structure may also support more detailed investigations into how different patterns of digital engagement relate to academic performance, mental health outcomes, stress, motivation, and student adjustment within higher education contexts.

The scale may also be useful for identifying patterns of digital engagement that deserve closer attention. For example, high levels of Digital Dependence and Frustration or Task Interference may point to areas where students are struggling to cope with digital demands. Although the DWS is not a diagnostic instrument, it can help highlight domains where early support or preventive action may be helpful. Within university settings, this may assist student support services and mental health professionals in promoting healthier digital habits and stronger emotional self-regulation. This may be particularly important given growing concerns within the well-being literature regarding stress, emotional exhaustion, attentional difficulties, and psychological strain associated with intensive digital engagement among young adults (34, 35). The scale may additionally serve as a screening or monitoring tool for identifying students who may be vulnerable to problematic digital engagement, emotional strain, or difficulties balancing academic and digital demands.

The scale may further support the design of interventions. As digital technologies continue to shape learning and communication, institutions are under increasing pressure to promote more sustainable patterns of digital use. The DWS could help identify the areas in need of support, whether that involves attentional control, emotional regulation in digital settings or students’ sense of autonomy in their use of technology. Importantly, the multidimensional nature of the scale allows interventions to be more targeted rather than adopting a one-size-fits-all approach to digital well-being. For example, students experiencing high task interference may benefit from attentional management strategies, whereas students reporting elevated digital dependence and frustration may require support focused on self-regulation, coping skills, or digital boundary-setting.

More broadly, the findings reinforce the idea that digital engagement is now an important context for understanding psychological well-being. Digital environments are no longer marginal to students’ lives; they are woven into academic, social, and personal functioning. For that reason, having valid tools to assess digital well-being may be useful not only for research, but also for early support efforts aimed at reducing digital strain and promoting healthier patterns of technology use. The findings may also inform institutional policies and digital literacy initiatives aimed at fostering healthier and more balanced technology use within higher education environments. As universities increasingly rely on digitally mediated teaching, communication, assessment practices, understanding students’ digital well-being may become an important component of student support, academic engagement, and broader mental health promotion efforts.

### 5.2 Limitations and future directions

Several limitations associated with this study are worth noting. First, the study focused on university students in Ghana. While this focus was appropriate for an initial scale-development study, it also limits how far the findings can be generalised without further validation in other contexts. In addition, participants were drawn from a single public university, which may not fully reflect the diversity of digital experiences across higher education institutions within Ghana or other cultural and educational settings (36). Future studies should examine the psychometric properties of the DWS across multiple institutions, regions, and cultural contexts to establish broader external validity and cross-cultural applicability (37).

Second, the study relied on self-report data. Although self-report measures are common in behavioural research, they are open to memory, perception, and response biases. Students may therefore under-report or over-report aspects of their digital behaviour. Social desirability bias and subjective interpretations of digital experiences may also have influenced participants’ responses. Future research could strengthen the assessment of digital well-being by incorporating complementary data sources such as behavioural usage indicators, digital activity logs, peer reports, or longitudinal digital tracking measures.

Third, the cross-sectional design does not allow conclusions about change over time. The study was designed to examine the psychometric properties of the scale rather than the developmental course or causes of digital well-being. Longitudinal work would therefore be useful. Future longitudinal and prospective studies could help determine how digital well-being develops over time and whether dimensions, such as task interference, emotional regulation, or digital dependence predict later psychological, academic, or behavioural outcomes. Such designs may also provide stronger evidence regarding the directional relationships among the dimensions of digital well-being and related psychosocial factors.

Additionally, although the Digital Dependence and Frustration dimension demonstrated acceptable psychometric performance, it contained fewer items and comparatively lower reliability estimates than the other dimensions. Future studies may therefore consider expanding and further validating this subscale across different populations and cultural contexts.

Finally, invariance testing was limited to gender. Other potentially important comparisons, such as programme of study, level of study, socioeconomic background or intensity of technology use, were not examined and should be considered in future research. Although configural and metric invariance were supported across gender groups, the weaker support for scalar invariance suggests that some items may function differently for male and female students. This limits the extent to which direct mean-level comparisons across gender groups can be interpreted unequivocally. Future studies should therefore investigate potential item-level sources of non-invariance or revised item formulations may improve comparability across demographic groups. Additional invariance testing across other relevant subgroups and cultural contexts would further strengthen the generalisation and interpretability of the DWS.

## 6 Conclusion

This study developed and validated the DWS among university students and contributes to the growing digital mental health literature by providing a multidimensional measure of students’ experiences within technology-mediated environments. The study supports the view that digital well-being is a multidimensional construct that extends beyond narrow indicators such as screen time or problematic technology use. Instead, students’ digital well-being appears to reflect a complex interplay of attentional, emotional, behavioural, regulatory, and balance-related experiences associated with everyday digital engagement.

The findings further suggest that digital well-being is closely connected to broader aspects of psychological functioning, including emotional regulation, perceived control, and digital strain, and the ability to maintain healthy patterns of engagement within digitally intensive environments. In this respect, the DWS offers a more contextually sensitive understanding of digital well-being that reflects the realities of contemporary university students’ academic and personal digital lives. Overall, the DWS provides a useful foundation for future research examining digital engagement and psychological functioning among university students.

## Data Availability

The raw data supporting the conclusions of this article will be made available by the authors, without undue reservation.
