# Supplementary material for: Development and validation of the digital well-being scale using university students
Source: Front Psychiatry. 2026 Jul 17;17:1853998. doi: 10.3389/fpsyt.2026.1853998 (PMC13424425; doi:10.3389/fpsyt.2026.1853998)
Supplement: Supplementary file 1 [file SupplementaryFile1.docx]

**Supplementary Information**
